# Molecular Biomarkers for the Diagnosis and Prognostication of Pancreatic Ductal Adenocarcinoma

**DOI:** 10.3390/jpm15060236

**Published:** 2025-06-05

**Authors:** James Sun, Morcos A. Awad, Jennifer Hwang, Anthony M. Villano

**Affiliations:** 1 Department of Surgical Oncology, Fox Chase Cancer Center, 333 Cottman Ave., Philadelphia, PA 19111, USA; james.sun@tuhs.temple.edu (J.S.); jennifer.hwang@fccc.edu (J.H.); 2 Department of General Surgery, Geisinger Health System, 100 N. Academy Drive, Danville, PA 17822, USA; maawad1@geisinger.edu

**Keywords:** pancreatic ductal adenocarcinoma, pancreatic cancer, personalized medicine, biomarkers, targeted therapy, molecular diagnostics, liquid biopsy

## Abstract

Pancreatic ductal adenocarcinoma (PDAC) remains among the most aggressive malignancies in the United States. Advances in treatments have slowly increased survival rates; however, outcomes remain dismal, largely due to the insidious onset of the disease and lack of screening tests leading to diagnosis at more advanced disease stages. As we better understand the molecular mechanisms that drive PDAC, we can leverage this technology for early detection of new PDAC or recurrences and find more effective methods to track treatment response. Liquid biopsies are increasingly common for the treatment of many malignancies, leveraging better technology to detect scant quantities of circulating tumor cells (CTCs) or byproducts of tumor biology (e.g., exosomes and microRNA [miRNA]) in the blood stream. When combined with existing biomarkers like CA 19-9, there is promising research that improved diagnostic modalities may be available in the future. Furthermore, these technologies are being leveraged to better prognosticate patients with PDAC and potentially monitor treatment responses not captured by cross-sectional imaging, which may allow for real-time changes in therapeutic strategy. This manuscript will review the molecular mechanisms that drive PDAC development and the biomarkers available for diagnosis and prognostication. Much of the data presented is still investigational, though many trials are ongoing to translate these studies for clinical use.

## 1. Introduction

Pancreatic ductal adenocarcinoma (PDAC), which accounts for 90% of all pancreatic cancers, is among the most aggressive malignancies and is the third leading cause of cancer-related deaths in the United States, with a five-year survival rate of 13% [1,2]. The poor prognosis of PDAC is related to numerous factors, including late stage of presentation, genomic instability, inter- and intra-tumoral biological heterogeneity, resistance to therapies, and high metastatic potential [3,4]. The lack of symptoms in early-stage disease contributes to this poor prognosis, given that about 50% of patients present with metastatic disease and only 15–20% present with a surgically resectable tumor [5]. A recent Surveillance, Epidemiology, and End Results (SEER) study by Blackford et al. identified an increase in the proportion of patients diagnosed at earlier stages and a younger age, potentially a reflection of high-risk screening programs and improved access to healthcare [6]. They reported a significant improvement in five-year overall survival (OS) of stage IA PDAC to 83.7%, although the lack of a reliable screening test makes early diagnosis challenging.

Multimodality treatment with surgical resection and cytotoxic chemotherapy remains the standard for localized disease at presentation. Improvements in PDAC survival are largely attributable to more effective chemotherapy regimens, with much debate regarding the optimal treatment sequencing. Current guidelines recommend adjuvant FOLFIRINOX (5-fluorouracil, oxaliplatin, leucovorin, and irinotecan) or gemcitabine plus nab-paclitaxel due to the improvement in progression-free (PFS) and overall survival (OS) extrapolated from trials in the metastatic space [7,8,9]. Among patients with anatomically borderline-resectable disease (and select patients with anatomically resectable disease) neoadjuvant therapy is recommended prior to surgical resection. At advanced stages, chemotherapy is the only remaining treatment option with low overall response rates.

Even with such advances in the multimodal therapeutic approach to PDAC, the prognosis is dismal due to the challenges in establishing a diagnosis at early stages of disease. At present, no biomarker has been identified that allows for early diagnosis of PDAC. Ideally, a screening test that would allow for early detection or even prevention of PDAC is desperately needed and represents an area of extreme interest in the field. The majority of patients are not surgical candidates at the time of diagnosis; therefore, identification of a biomarker for early diagnosis would afford more patients the opportunity to undergo curative resection.

**Figure 1:**
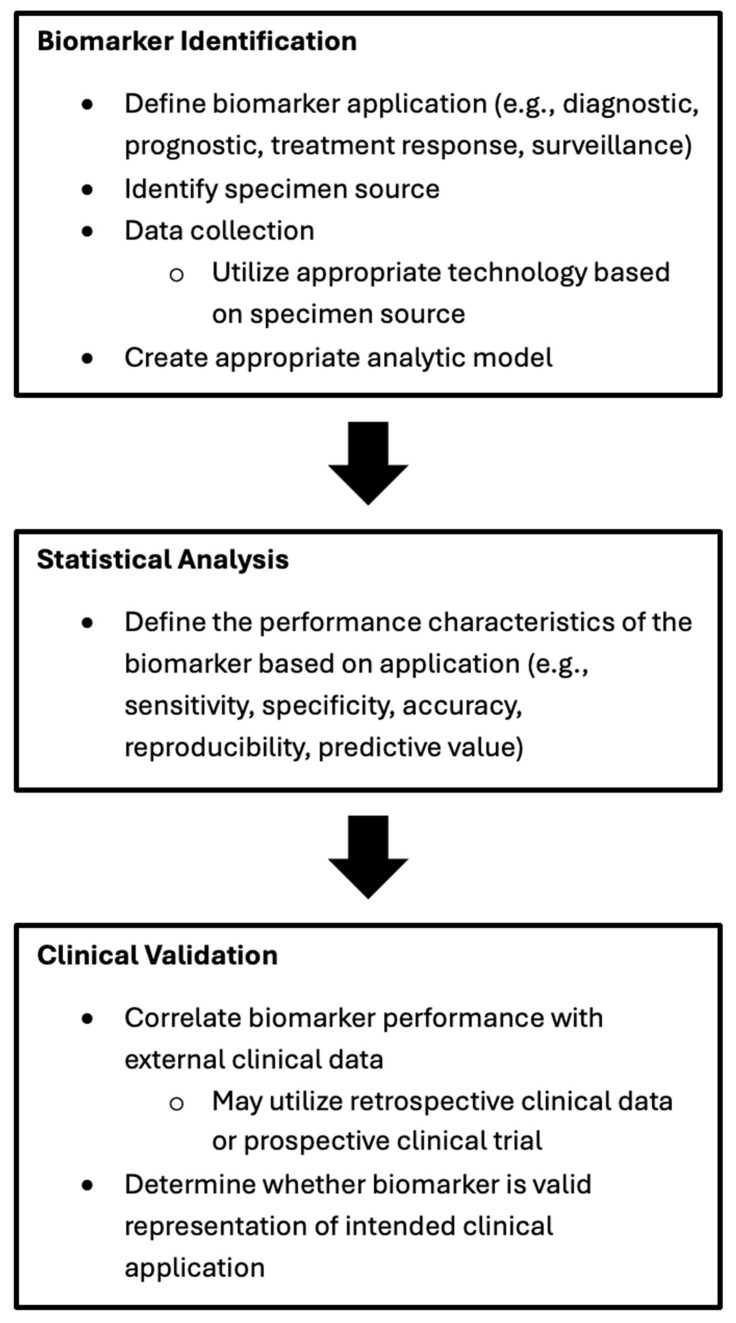
Schematic overview of biomarker discovery steps.

Biological markers, or biomarkers, are “a defined characteristic that can be measured as an indicator of normal biological processes, pathogenic processes, or biological responses to an exposure or intervention, including therapeutic interventions” [10]. Clinical paradigms are shifting towards using molecular biomarkers obtained directly from patients to personalize cancer care. Biomarkers have been utilized at several timepoints in a patient’s cancer treatment, including diagnosis (detection), prognostication, predicting treatment response, and surveillance for disease recurrence or progression. Figure 1 highlights the steps involved in biomarker identification to statistical analysis and then clinical validation.

Herein, we will review the crucial genetic alterations that drive PDAC and provide novel therapeutic targets in the era of personalized medicine. We will discuss the biomarkers utilized for the management of PDAC, most of which are focused on diagnosis and prognostication. These biomarkers, including serum proteins, cell-free DNA (cfDNA), circulating tumor DNA (ctDNA), cell-free RNA (cfRNA), circulating tumor cells (CTCs), and exosomes, are summarized in Table 1. Lastly, we will discuss the cost-effectiveness of molecular profiling for the identification of novel therapeutic targets.

## 2. Genetic Biomarkers

The latest National Comprehensive Cancer Network (NCCN) guidelines currently recommend that all patients diagnosed with PDAC and their first-degree relatives receive germline mutation testing to identify actionable mutations for targeted therapy and clinical trial participation [11,12]. The Know Your Tumor (KYT) program was an initiative that provided molecular profiling for PDAC patients in the United States to identify targetable mutations to tailor individualized treatment strategies for patients [13]. Between 2014 and 2019, 1856 patients were enrolled, of which 58% of patients received molecular testing results; actionable mutations were identified in 26% of these patients. Patients who received targeted therapy based on molecular profiling had significantly improved median OS compared to patients who received standard therapy (median OS 2.58 vs. 1.51 years, *p* = 0.0004) [13]. These findings highlight the importance of molecular testing to identify personalized treatment targets, which have tangible OS benefits.

Additionally, a thorough family history is warranted and any patient with clinical suspicion for inherited susceptibility or hereditary cancer syndrome should be referred for further genetic testing and counseling [14]. While there is currently no screening protocol, genetic biomarkers could play a role in identifying patients at high risk for developing PDAC to implement a screening protocol with hopes of early diagnosis, thereby improving prognosis [15].

### 2.1. Oncogenes

Activating mutations in the KRAS oncogene are identified in up to 96% of patients with PDAC [16,17,18]. KRAS is a proto-oncogene that acts as an activating kinase by phosphorylating its inactive GDP-containing state to an active GTP-containing state. KRAS signals multiple downstream pathways (including RAF/MEK/ERK and PI3K/PDK1/AKT), which upregulate proliferation and promote oncogenesis [19].

Three common single-point mutations at G12, G13, and Q61 cause constitutive activation of KRAS [20]. The most common are the point mutations G12D (36% of mutations) and G12V (35% of mutations), followed by G12R (13% of mutations) and G12C (1.3% of mutations). Patients with the G12C mutation had the shortest median OS (8 months), while G12R mutations have been associated with longer median OS (13.2 months) [21]. In patients with locally advanced or metastatic PDAC, the *KRAS* G12D mutation was found to be a negative predictor of OS when compared to other G12 mutations [22].

*KRAS* G12C is the only mutation with approved selective targeting inhibitors, such as adagrasib and sotorasib [23,24]. In a clinical trial investigating sotorasib as a second-line monotherapy in *KRAS* G12C-mutated pancreatic cancer, 21% of patients experienced a partial response [24]. The median PFS (4.0 months) and OS (6.9 months) of patients receiving sotorasib were longer than those for other second-line regimens [24]. One of the challenges with targeting *KRAS* G12C is drug resistance, especially with monotherapy [23,25,26]. While resistance mechanisms are not fully understood, their multifaceted and evolutionary nature call for the development of combination therapies [26]. As G12C mutations are uncommon in PDAC, there is much interest in the development of G12D inhibitors. Preclinical data demonstrating the efficacy of the G12D inhibitor MRTX1133 has informed an ongoing phase 1/2 study, which is expected to accrue in 2025 (NCT05737706) [27]. In clinical practice, G12C inhibitors (sotorasib and adagrasib) are approved for use in the metastatic setting after progression through first-line therapeutic options in patients harboring this point mutation.

### 2.2. Tumor Suppressor Genes

Mutations in *TP53* are found in up to 80% of PDAC cases [28,29]. Mutations are categorized into deletions, which result in absence of the TP53 protein or, more commonly, point mutations, which result in a gain of function [30]. Loss of the TP53 protein results in uncontrolled cell division, whereas a point mutation forms a stable mutant protein that promotes proliferation and has been shown to cause chemotherapy resistance [29]. The absolute risk of developing PDAC in patients with a *TP53* mutation is about 5%. *TP53* point mutations have been shown to accelerate metastasis and a meta-analysis showed a correlation with shorter OS (hazard ratio [HR] 1.249, *p* = 0.047) [30,31].

*SMAD4* is a gene that encodes a transcription factor that helps regulate the TGF-β pathway [29,30]. A mutation or loss of this gene promotes disease progression by dysregulating autophagy, invasion, and metastasis. *SMAD4* inactivation is prevalent in up to 60% of PDAC cases [31,32]. The prognostic value of *SMAD4* is not well understood. A meta-analysis by Gu et al. demonstrated an association between loss of *SMAD4* expression and worse OS (HR 1.397, *p* = 0.04). A study by Dardare et al. found an association with worse disease-free survival, but not OS [32].

*CDKN2A* is a gene involved in cell proliferation and encodes proteins p16 and p14 [33]. Mutations can lead to dysregulation in apoptosis and invasion. Patients with *CDKN2A* mutations carry an absolute risk of greater than 15% for developing PDAC and the NCCN practice guidelines recommend PDAC screening for these patients [34]. Higher expression of wild-type *CDKN2A* has been linked to improved prognosis, but mutations in *CDKN2A* have not been associated with changes in OS [29,31]. Palbociclib, a CDK4/6 inhibitor, has been studied as a targeted therapy in CDKN2A-mutated PDAC patients. However, 16 weeks of palbociclib monotherapy did not show a significant response [35]. There are several ongoing clinical trials investigating whether combining CDK4/6 inhibitors with other types of therapies produces a synergistic effect [33].

*STK11* mutations cause dysregulation in cell proliferation and DNA damage response. Mutant carriers of *STK11* carry an absolute risk of up to 36% of developing PDAC [36]. PDAC screening is recommended for all patients with a *STK11* germline mutation [34]. No novel targeted therapeutic agents have been identified for these mutations.

### 2.3. DNA Damage Repair Genes

*BRCA1*, *BRCA2*, and *PALB2* are necessary components for the *RAD51*-dependent homologous recombination pathway of double-stranded DNA repair [37]. Inhibition of these genes causes failure to repair DNA strands. *BRCA* mutations occur in about 5.9–7.2% of PDAC patients [37]. The risk of developing PDAC is 5–10% for *BRCA2* carriers and 1–5% for *BRCA1* [34,38]. In PDAC patients who received surgery, *BRCA2* was associated with improved OS when compared to patients with *BRCA1* (HR 0.64, *p* = 0.02), with patients receiving platinum-based treatment experiencing increased benefit [39].

Mutations in *PALB2*, a *BRCA*-interacting protein, are the second most common cause of hereditary PDAC. Patients with these mutations carry a 2–5% risk of developing PDAC [34,40]. *PALB2* mutations are found in 3–4% of familial PDAC cases and have been identified as a PDAC susceptibility gene.

Homologous recombination-deficient cells, such as tumor cells containing a *BRCA* mutation, utilize poly ADP-ribose polymerase (PARP) for DNA repair through alternative end-joining [41,42,43,44,45]. Through synthetic lethality, recent studies have demonstrated the efficacy of PARP inhibitors in causing the death of cells with *BRCA* mutation [46]. The POLO phase 3 trial showed a response rate of 23% to Olaparib, compared to 12% with placebo, as maintenance therapy after platinum-based chemotherapy among *BRCA*-mutated patients with metastatic PDAC [47]. While there was no statistically significant OS benefit, patients treated with Olaparib experienced clinically significant benefits, including a well-tolerated safety profile and longer time without chemotherapy [48]. In other reports, PARP inhibitors have been used in patients with *BRCA* and/or *PALB2* germline mutations, with response rates ranging from 20 to 70% depending on the setting of use, whether in the case of progression or maintenance after standard therapy [49,50,51,52]. In current practice, PARP inhibitors are recommended as maintenance therapy in *BRCA*-mutated, metastatic PDAC with no disease progression within 4–6 months of first line platinum-based chemotherapy.

*ATM* produces a serine/threonine kinase that is essential for repairing double strands of DNA [53]. The absolute risk for developing PDAC among *ATM* variant carriers can be as high as 10%. *ATM* mutations are prevalent in up to 6% of patients with PDAC. In one study by Hannan et al., patients with *ATM* mutation and PDAC had improved OS when compared to the wild-type ATM group (40.2 months vs. 15.5 months, *p* = 0.001) [54].

### 2.4. DNA Mismatch Repair Genes

The mismatch repair (MMR) gene family, including *MLH1*, *MSH2*, *MSH6*, and *PSM2*, is involved in base–base mismatch repairs during DNA replication. Mutations in this family of genes result in high microsatellite instability (MSI-H) resulting from accumulation of DNA errors [55]. In MSI-H tumor cells, which are MMR-deficient (dMMR), these mutations lead to the development of numerous unchecked errors in DNA replication, yielding neo-antigens, which are recognized by the immune system and presented in major histocompatibility complex class I molecules found on cytotoxic T-cells. In this setting, blocking the binding of PD1 on T-cells to PDL1 on tumor cells using checkpoint inhibitors, such as pembrolizumab, an anti-PD1 antibody, causes T-cell activation and targeting of tumor cells and the tumor microenvironment [56]. MMR mutation or deficiency only occurs in 1–2% of PDAC patients [37].

The KEYNOTE-158 study was a phase 2 trial that enrolled patients with previously treated, non-colorectal MSI-H/dMMR tumors, including PDAC, which accounted for 6.3% of the study cohort (n = 351). Patients received pembrolizumab every 3 weeks for a total of 35 weeks, or until disease progression or unacceptable toxicity. Among all patients, the objective response rate (ORR) was 30.8% (95% CI 25.8–36.2%), with a complete response (CR) rate of 8.4% with a median follow-up of 37.5 months [57,58]. Among PDAC patients, the ORR was 18.2% (95% CI 5.2–40.3%) and CR rate was 4.5% (1/22 patients). The duration of response and 3-year PFS rate were not reached, suggesting there is continued treatment efficacy, though the results of a small cohort single-arm study should be interpreted with caution.

Despite the promising survival data, mismatch repair deficiency is rare in PDAC and immunotherapy may hold promise for a very small proportion of patients as a single-agent strategy. As PDAC is generally considered an immunologically cold tumor, research exploring how to render the microenvironment susceptible to immunotherapeutic approaches is direly needed given clinical responses are robust when responses are seen.

## 3. Liquid Biomarkers

### 3.1. Carbohydrate Antigen 19-9 (CA 19-9)

#### 3.1.1. CA 19-9 for Pancreatic Cancer Screening

Serum CA19-9 (carbohydrate antigen 19-9 or sialyl Lewis A) has been associated with PDAC for decades and is most frequently used for staging and surveillance. CA19-9 is produced throughout the gastrointestinal tract, including the pancreas, biliary system, stomach, colon, uterus, and salivary glands [59]. As a result, it is elevated for a range of conditions, both benign and malignant, thus limiting its role as a screening biomarker for PDAC detection. Kim et al. measured CA 19-9 levels in 70,940 asymptomatic patients [60]. Only 1063 (1.5%) of these had elevated CA 19-9 levels, of which 4 patients were diagnosed with PDAC. Thus, the positive predictive value in the asymptomatic population was 0.9%, with a sensitivity of 100% and specificity of 98.5%. Among symptomatic patients (epigastric pain, weight loss, and jaundice), the sensitivity for CA19-9 ranged from 79 to 81%, with a specificity of 82–90%, though the positive predictive value was only 0.5–0.9%, limiting its utility as a screening test [61,62,63]. In addition, some patients with PDAC have documented CA19-9 levels within normal limits, while the rate of false-positive elevation in CA19-9 has been reportedly high as well. Only 80% of patients with PDAC will have elevated CA19-9 levels.

To further complicate the issue, CA 19-9 expression requires the presence of the fucosyltransferase 3 (FUT3) enzyme, which is produced by people with Lewis antigen-positive blood types. Approximately 10% of the population do not produce CA 19-9 [64]. Therefore, a falsely normal CA19-9 level further limits its use for screening [65]. Recent research has identified a tumor marker gene test to improve the diagnostic performance of CA 19-9 [66]. Instead of simply understanding CA 19-9 non-secretion as a binary function, four genetic groups have been identified to best characterize CA 19-9 expression based on the presence of *FUT3* and *FUT2* gene variants (e.g., FUT3-null individuals produce no CA 19-9 and FUT2-null individuals with at least one *FUT3* allele produce the most CA 19-9) [65,66,67]. Reference ranges for CA 19-9 in each of these categories have been identified, thus improving its diagnostic performance amongst enzymatic variants [66].

Duke pancreatic monoclonal antigen type 2 (DUPAN-2 or sialyl Lewis C) is the immediate precursor to CA 19-9, converted by FUT3 [68]. It is also elevated in patients with PDAC and more frequently used in Japan to monitor disease burden, especially in patients who do not produce CA 19-9 [69,70]. DUPAN-2 levels are affected by the same *FUT* variants that affect CA 19-9 synthesis, introduced above. Ando et al. recently characterized three functional groups by expression of *FUT3* and *FUT2,* with significant differences in DUPAN-2 levels [71]. Again, reference ranges for DUPAN-2 were established for each category, thereby improving the diagnostic performance. Furthermore, combining DUPAN-2 and CA 19-9 alongside FUT classification resulted in the best diagnostic performance, with 62% sensitivity, 98% specificity, and area under the curve (AUC) 0.919 among stage I PDAC cases and AUC 0.960 for patients with stage I or II PDAC [65,71]. This high diagnostic accuracy warrants further investigation for use as an early detection test.

While CA 19-9 alone has proven to be a poor diagnostic test, there are many studies of CA 19-9 in combination with other biomarkers that have demonstrated more promising diagnostic results, though none are presently approved for clinical use. Metalloproteinase-1 (TIMP-1) and Leucine-rich alpha-2-glycoprotein 1 (LRG1) have previously been identified as complementary tests to CA 19-9 level when measured together [72]. TIMP1 has been found to increase tumor proliferation and LRG1 promotes angiogenesis through VEGF pathway activation [72,73]. Both are found in cancers aside from PDAC. A recent study by Ben-Ami et al. found that the combination of all three protein markers increases the sensitivity and AUC of CA19-9 at 95% and 98% specificity in patients with early-stage PDAC compared with healthy individuals and patients with chronic pancreatitis [74,75].

#### 3.1.2. CA 19-9 for Pancreatic Cancer Prognostication

There is extensive literature to support CA 19-9 as a prognostic biomarker at each phase of PDAC treatment. Lower levels at diagnosis [76,77] and after resection [78] have been associated with improved OS compared to higher levels. Even among patients with advanced disease, lower CA 19-9 levels again correlate with better response to systemic therapy [63]. CA 19-9 levels have also been shown to correlate well with pathologic stage, increasing with advancing stage [79]. CA 19-9 levels also correlate with resectability, with lower levels among patients with negative occult metastatic disease during laparoscopy compared to those found to be unresectable [80,81]. CA 19-9 levels are frequently used to assess treatment response after neoadjuvant therapy, during adjuvant treatment, and for surveillance after resection. Decreases in CA 19-9 have been correlated with survival after neoadjuvant therapy [82] and after resection [83] and are predictive of response to systemic therapy among patients with advanced disease, thus rendering it a useful marker to inform treatment responses [84]. Accordingly, a persistently elevated CA 19-9 level post resection has been associated with worse survival and higher recurrence rates [85]. Rising CA 19-9 levels have been shown to precede imaging findings in the setting of recurrent disease [86,87].

In summary, although CA 19-9 levels have not been demonstrated to be an effective screening tool, both absolute levels and changes in levels once diagnosed (in producers of this biomarker) reflect disease status and are a useful surrogate for disease burden upon diagnosis and assessment of treatment response after neoadjuvant therapy and resection. A rising CA 19-9 level should raise suspicion for recurrent disease and often precedes imaging findings.

In the clinical setting, absolute CA19-9 levels as well as relative change while on therapy inform decisions surrounding the utilization of neoadjuvant therapy, surgical resection, and sometimes the implementation of additional locoregional therapy such as radiation. An increase in CA19-9 may also inform a change in chemotherapy in the metastatic setting. Practices vary widely in this regard and there are no guidelines that provide specific recommendations for how oncologists should alter management based on CA19-9. However, failure of CA19-9 to decrease while receiving treatment has generally been viewed as a poor prognostic indicator and recently has informed several studies using this as a criterion for switching the chemotherapy regimen between the two currently approved regimens (FOLFIRNOX or gemcitabine and nab-paclitaxel) with favorable results [88,89,90]. We anticipate that similar strategies implementing CA19-9 in clinical decision-making will continue to grow as we expand approved therapies for localized PDAC, thus personalizing the approach based on individual patient responses to treatment.

### 3.2. Human Epidermal Growth Factor Receptor 2 (HER2)

Human epidermal growth factor receptor 2 (HER2) was first recognized as an oncogene contributing to the pathogenesis of breast cancer and is a well-established therapeutic target for breast and an increasing number of gastrointestinal cancers [91]. Activation of HER2 leads to signal transduction, resulting in cell division, differentiation, and survival. Advances in molecular profiling have identified HER2 overexpression across various tumor types, including gastric, gastroesophageal junction, colorectal, lung, and pancreatic cancers [92]. HER2 overexpression has been inconsistently reported among PDAC, with a broad range of expression from 0 to 82% of patients; more recent studies suggest that the incidence is closer to approximately 40% [93,94,95,96].

Trastuzumab, a human recombinant anti-HER2 antibody, has revolutionized cancer treatment by improving survival for HER2-positive breast and gastric cancers [97,98]. Trastuzumab deruxtecan (T-DXd), a novel drug–antibody conjugate, is a recent development with demonstrated efficacy against tumors with low HER2 expression [93]. This has led to accelerated Food and Drug Administration (FDA) approval for the treatment of previously treated, unresectable, and metastatic solid tumors regardless of tumor type on the basis of the DESTINY-PanTumor02, Lung01, and CRC02 trials [99,100,101,102]. Among the 267 patients enrolled in this trial, pancreatic cancer patients made up 9.3% of the cohort (n = 25). Other tumor types enrolled included endometrial (n = 40), cervical (n = 40), ovarian (n = 40), bladder (n = 41), biliary tract (n = 41), and other cancers (n = 40). The first 10 pancreatic cancer patients did not respond to T-DXd and therefore the cohort was closed to further enrollment due to futility. Of the 25 pancreatic cancer patients analyzed in the study, the objective response rate (ORR) was 4%. Despite low response rates, patients often have few therapeutic options and T-DXd remains approved as subsequent therapy for HER2-amplified locally advanced or metastatic and recurrent PDAC unresponsive to first-line options [11].

### 3.3. Claudin 18.2

Claudins are transmembrane proteins and one of the major components of tight junctions, which regulate cellular permeability [103,104]. There are presently 27 known CLDN proteins with different expression patterns that have been associated with different cancers depending on the primary tumor site [105]. Under normal physiological circumstances, Claudin 18.2 is only expressed in gastric mucosa [106]; however, it is frequently overexpressed in gastric, esophageal, pancreatic, and ovarian adenocarcinomas [107].

#### 3.3.1. Claudin 18.2 as a Therapeutic Target

Zolbetuximab, an immunoglobulin G1 monoclonal antibody targeting Claudin 18.2, received FDA approval in 2024 as a first-line treatment for locally advanced unresectable or metastatic HER2-negative gastroesophageal junction and gastric tumors that express Claudin 18.2 in combination with chemotherapy on the basis of two phase 3 clinical trials [108]. Both the SPOTLIGHT [109] and GLOW [110] trials reported significantly improved PFS (10.6 vs. 8.7; 8.2 vs. 6.8 months, respectively) and OS (18.2 vs. 15.5; 14.4 vs. 12.2 months, respectively) with combination treatment compared to chemotherapy alone. There is an ongoing phase 2 clinical trial investigating patients with metastatic PDAC and high Claudin 18.2 expression comparing combination zolbetuximab and chemotherapy (gemcitabine and nab-paclitaxel) versus chemotherapy alone (NCT03816163) [111]. This study is still accruing, and the results are eagerly awaited. At present, there are no published studies reporting the efficacy of zolbetuximab for PDAC and it is not yet an approved therapeutic option.

#### 3.3.2. Claudin 18.2 as a Pancreatic Cancer Biomarker

Specific to PDAC, a study by Wöll et al. analyzed Claudin 18.2 expression in normal pancreatic tissue (n = 24), known PDAC (n = 202), and metastatic PDAC tissue (n = 84) [112]. None of the normal pancreatic tissue expressed Claudin 18.2. Among tested PDAC tissue, 59.2% of primary tumors, 69.4% of metastatic lymph nodes (n = 34), and 65.7% of liver metastases (n = 23) expressed Claudin 18.2. Wang et al. performed a similar study, comparing Claudin 18.2 expression between normal pancreatic tissue and known PDAC [113]. They confirmed significantly higher Claudin 18.2 expression in PDAC tissue, though this did not correlate with better OS. The authors then also analyzed Claudin 18.2 expression in PDAC and pancreatic tissue adjacent to a tumor and reported positive expression in 94.6% and 94.2% of specimens, respectively. Lastly, the authors reported that Claudin 18.2 expression correlated with higher disease stage, lymph node and distant metastases, nerve invasion, and worse OS.

Most recently, Lyu et al. again assessed Claudin 18.2 expression in PDAC tissue, specifically utilizing the VENTANA CLDN18 (43-14A) assay and the positivity definition (≥75% tumor cells with immunohistochemistry staining intensity ≥2+) used in the SPOTLIGHT and GLOW trials [105,109,110,114]. Contrary to the previous studies, Claudin 18.2 expression was identified among 30.4% of specimens. The authors also report significantly improved OS among patients with Claudin 18.2 expression compared to patients with negative expression (median OS 30 vs. 18 months, *p* = 0.003). The authors explain that these findings are due to alignment with the stricter definitions used in published clinical trials.

## 4. Circulating Tumor Cells

Circulating tumor cells (CTCs) are cells isolated from peripheral circulation and are released from primary and/or metastatic epithelial tumor sites. CTCs provide a comprehensive picture of the biology of a tumor, including the differing surface-antigen profiles and multiple single-cell genomic expression profiles. For this reason, CTCs may assist in identifying targeted therapies for patients with PDAC. Other clinical applications include cancer diagnosis, prognostication, treatment monitoring, and detection of recurrence [115]. CTCs are rare in circulation, leading to different strategies used to isolate these cells for analysis. The number of CTCs varies widely when reviewing the current literature, ranging from as low as 1 cell per 10^9^ blood cells to 10–100 cells per 10^6^–10^8^ cells [116,117]. The technologic details of CTC enrichment and identification will not be discussed in detail in this manuscript. At present, the only FDA-approved method for isolating CTCs is CellSearch^®^ (Huntingdon Valley, PA, USA), utilizing epithelial and tumor surface antigens. CTCs can also be found in patients with high-risk pancreatic lesions such as mucinous cystic neoplasms (MCNs) and IPMNs [118].

### 4.1. Circulating Tumor Cells for Pancreatic Cancer Diagnosis

Some studies have explored the possibility of using CTCs for PDAC diagnosis. Rhim et al. hypothesized that PDAC cells could be detected in circulation prior to tumor formation [119]. Circulating epithelial cells (CECs), circulating cells of pancreatic epithelial cell origin as opposed to tumor origin (CTC), were identified in patients with precancerous pancreatic cystic lesions, cytology-confirmed PDAC, and a control group of average-risk adults presenting for age-appropriate colonoscopy screening. Interestingly, 8/20 (40%) patients with cystic pancreatic lesions had detectable CECs. None of the patients in the control group and 7/9 (78%) PDAC patients had detectable CECs. Although clinical follow-up of patients in this cohort is not available, this was the first study to demonstrate early identification of circulating cells in patients with precancerous pancreatic lesions, suggesting that cancer cells can be isolated in circulation prior to a detectable pancreatic tumor. Another study by Ankeny et al. specifically measured CTCs obtained at the time of new PDAC diagnosis with a novel method of detection using a NanoVelcro platform [120]. CTCs were identified in 54/72 patients with confirmed PDAC. When ≥1 CTC was identified per 4 mL of venous blood, sensitivity was 75% and specificity was 95.7% for PDAC diagnosis. Furthermore, detection of ≥3 CTC/4 mL venous blood differentiated between local/regional and metastatic disease. Buscail et al. performed a prospective trial evaluating the diagnostic performance of identifying CTC for PDAC diagnosis in patients with early-stage resectable PDAC [121]. They found that combining CTC and GPC1-positive exosome detection yielded a diagnostic sensitivity of 100%, specificity of 80%, and negative predictive value (NPV) of 100%, far superior in comparison to established diagnostic paradigms using endoscopic ultrasound (EUS)-guided biopsy and serum CA 19-9 (50% sensitivity, 92% specificity, and 70% NPV). At present, CTC detection techniques are still a limiting factor; however, early studies suggest that one-day liquid biopsy for CTCs may be an adjunct for early detection of PDAC.

### 4.2. Circulating Tumor Cells for Pancreatic Cancer Prognostication

In terms of PDAC prognostication, cross-sectional imaging routinely understages patients, leading to high postoperative recurrence rates due to occult metastatic disease below the detection threshold of modern imaging techniques. Building on their previous study, Court et al. studied whether preoperative CTC detection was associated with occult metastatic disease [122]. In a group of 126 patients across all clinical disease stages and 26 patients with benign pancreatic masses, they demonstrated that a cutoff of ≥3 CTC/4 mL venous blood differentiated patients with occult metastatic disease from those with localized resectable PDAC with a sensitivity of 85%, specificity of 80%, PPV of 94%, NPV of 58%, and AUROC of 0.82 (*p* < 0.0001). This performed better than a CA19-9 cut-off of 500 U/mL, which yielded a sensitivity of 60.0%, specificity of 77.3%, PPV of 81.0%, NPV of 54.5%, and AUROC of 0.62 (*p* = 0.084). They also found that CTC count was associated with OS (HR 1.69), which remained a significant factor in multivariable analysis (HR 1.38, both *p* < 0.001). The authors concluded that preoperative CTC detection has potential clinical applications for identifying occult metastatic disease and is associated with worse OS. Other studies have reported similar findings. Among patients with resectable stage II PDAC, the presence of CTCs was associated with an increased rate of liver metastasis [123]. At any stage of PDAC, mainly stages I and II, higher numbers of CTCs were associated with worse PFS and OS, regardless of detection method [122,124,125,126,127]. The presence and higher count of CTCs were also associated with earlier recurrence [126,128]. CTCs have also been investigated in the neoadjuvant setting as a biomarker for treatment response. The CLUSTER prospective trial enrolled 200 consecutive patients with presumed PDAC, of which 136 received pancreatectomy; 79/136 (58%) received upfront surgery; and 57/126 (42%) received neoadjuvant chemotherapy and resection. The primary goal was to evaluate CTC dynamics (fluctuations in CTC level during treatment) and its relationship with disease status, with secondary goals of utilizing CTCs for predicting early recurrence and disease-specific survival and evaluating its utility as a biomarker for disease recurrence [128]. Surgical resection reduced total CTC levels for both chemotherapy-naïve patients and patients that received neoadjuvant chemotherapy alike. Chemotherapy-naïve surgical patients had a median of 11 CTCs/mL of blood (interquartile range [IQR] 6–15), which decreased to a median of 2 CTCs/mL of blood (IQR 1–4, *p* < 0.001) following resection. Neoadjuvant patients had a preoperative median of 7 CTCs/mL (IQR 3–10), which decreased to a median of 2 CTCs/mL of blood (IQR 1–4, *p* < 0.001). CTC levels prior to neoadjuvant therapy were not available to assess how levels may fluctuate prior to surgery. Patients with occult metastatic disease at the time of surgery also had significantly higher total CTC levels (median 20 CTCs/mL blood, IQR 12–24) compared to patients who successfully underwent resection (median 3 CTCs/mL blood, IQR 2–6, *p* < 0.001). Among 59 (44%) patients who had disease recurrence within 1 year of surgery, preoperative total CTCs were also higher compared to patients who did not have early recurrence (median 11 CTCs/mL blood [IQR 8–16] vs. 5 CTCs/mL blood [IQR 3–11], *p* < 0.001). Multivariable analysis demonstrated that all studied populations of CTCs were independently associated with early recurrence, while CA19-9 at a cut-off of 178 U/mL was not predictive of early recurrence. Interestingly, CTCs were identified as early as 2 months prior to imaging evidence of recurrence. The authors concluded that CTC dynamics represent response to treatment and further support CTC levels as a prognostic biomarker for early disease recurrence. Once again, CTC isolation remains a challenge, limiting broad clinical applications at this time.

## 5. Circulating Tumor DNA

Circulating free or cell-free DNA (cfDNA) is extracellular DNA found in circulation, the majority of which comes from healthy host tissues [129]. Extracellular DNA from tumor cells is termed ctDNA and is present in minute concentrations in circulation, which can be as low as 0.1% of cfDNA among early-stage or resected cancers and up to 10% of cfDNA in advanced-stage cancers [129]. A discussion of the technology for ctDNA isolation is outside the scope of this review; however, in general, detection of ctDNA is based on targeting genetic or epigenetic anomalies using digital polymerase chain reaction (PCR), next-generation sequencing (NGS), and deep sequencing (Seq) technologies. These methods are primarily based upon identifying *KRAS* mutations, present in approximately 90% of PDAC patients [130,131]. These technologies are reviewed in depth elsewhere [129,132]. Detection of ctDNA, termed liquid biopsy, may have further clinical applications for PDAC diagnosis and prognostication, including surveillance of recurrence and prediction of treatment response [133].

### 5.1. Circulating Tumor DNA for Pancreatic Cancer Diagnosis

Typically, PDAC diagnosis requires identification of a tumor on imaging studies, which then leads to a diagnostic procedure for tissue biopsy, which carries procedural risks and potential for false-negative results. Early detection of PDAC is further limited by the lack of screening for average-risk patients; therefore, patients are often not diagnosed until advanced stages where secondary symptoms have manifested from a growing tumor. Nearly 80% of patients are diagnosed at advanced disease stage, often due to lack of imaging resolution to clearly delineate a pancreatic mass [134]. Therefore, there is much interest in improving ctDNA detection methods to serve as an early diagnostic tool. Although studies have demonstrated good concordance between mutations detected in ctDNA and primary tumor tissue, there is less than 50% concordance in early-stage PDAC [135,136]. To date, ctDNA has not been demonstrated as a reliable method of PDAC diagnosis [133].

Two well-studied ctDNA biomarker tests for cancer diagnosis are CancerSEEK [137,138] and the multi-cancer methylated DNA panel by GRAIL [139]. CancerSEEK is a multimodality biomarker test combining PCR for detection of commonly mutated genes and protein biomarkers that may be elevated among cancer patients (e.g., CA 19-9 in PDAC) for the early diagnosis of multiple cancers, including PDAC [137]. The study reported a sensitivity of 72% and specificity of 99% for detection of stage I-III PDAC. Unfortunately, among early-stage PDAC patients, only 25–30% had detectable ctDNA. The PATHFINDER trial utilized an NGS test for genomic DNA methylation patterns unique to each cancer type [139]. Of 6600 tested patients, 1.4% had a positive test result, of which 35 patients ultimately had cancer (0.5%); 1 of these patients had PDAC. They concluded that this test has a high specificity of 99%, high NPV of 98.6%, but low PPV of 38%. A revised test is under trial in a follow-up study (PATHFINDER 2, NCT05155605).

### 5.2. Circulating Tumor DNA for Pancreatic Cancer Prognostication

From a prognostic standpoint, detecting mutant *KRAS* ctDNA is associated with worse OS and PFS and earlier disease recurrence with a higher burden of metastatic disease, regardless of whether ctDNA is identified preoperatively or postoperatively [115,140,141,142,143]. Pietrasz et al. demonstrated that patients with detectable ctDNA postoperatively had significantly worse DFS (4.6 vs. 17.6 months) and OS (19.3 vs. 32.2 months) than those with detectable ctDNA [144]. A more recent study compared ctDNA detection before and after chemotherapy treatment among patients with metastatic PDAC [145]. The authors found that ctDNA was only detected in 56.6% of patients. They also reported that PFS (3.66, 95%CI 1.54–8.67) and OS (4.72, 95%CI 1.22–18.20, both *p* < 0.001) were worse among patients with detectable ctDNA after chemotherapy (nonresponders) compared to those with undetectable ctDNA (responders). Interestingly, they did not find a statistically significant difference in PFS and OS between patients with undetectable pretreatment ctDNA compared to responders. The authors concluded that there is potential for ctDNA to be used as a prognostication tool for patients with metastatic PDAC, with the limitation that better methods of ctDNA detection are needed.

Additionally, ctDNA tests are able to identify different *KRAS* mutations, which have also identified genetic subtypes with worse outcomes. Hálková et al. demonstrated that the most common *KRAS* mutations in PDAC are G12V, G12D, and G12R [146]. They demonstrate that *KRAS* G12D mutations are associated with the worst median OS (101 days, 95%CI 80–600 vs. 210 days, 95%CI 161–602, *p* = 0.0166) compared to the group with other KRAS mutations. Other studies have demonstrated worse OS with the G12V mutation as well [147,148].

## 6. Exosomes

Exosomes are small extracellular vesicles ranging in size from 50 to 150 nm that are responsible for intercellular communication and homeostasis [149,150]. They are released by nearly all cells, including cancer cells, and play a role in maintaining the tumor microenvironment to promote cancer growth and metastasis [151,152]. Cancer-derived exosomes can be isolated from body fluids, leading to potential clinical applications. Cancer-derived exosomes have different components and contents compared to exosomes from normal cells [153]. There is particular interest in exosome components that are upregulated in patients with PDAC, including proteins, microRNA (miRNA), long noncoding RNA (lnRNA), mRNA, and circular RNA (circRNA) [153]. There are many practical challenges in isolating exosomes due to their size and heterogeneity; however, ultracentrifugation is the most commonly used technology, which sorts exosomes based on mass and size though may disrupt exosome structure. The various detection methods are discussed in further detail in other review manuscripts [154,155].

From a clinical perspective, exosome detection is under investigation as a diagnostic tool. Advantages include isolation from fluid sources other than peripheral blood, such as serum, plasma, pancreatic fluid, and saliva [156]. Detection of some exosomal miRNAs, such as miR-21, miR-210, miR-10b, miR-3976, and miR-1246, have been correlated with early detection of PDAC [156,157,158,159]. Additionally, miRNA detection in conjunction with CA 19-9 levels has been demonstrated to have both diagnostic and prognostic value. Reese et al. demonstrated that among patients with PDAC, miR-200b and miR-200c were overexpressed compared to normal healthy patients and those with pancreatitis [160]. When combined with CA 19-9, this biomarker panel had a diagnostic accuracy of 97%. Expression of these miRNAs was correlated with shorter OS. Similarly, Guo et al. demonstrated that serum ratios of miR-95-3p to miR-26b-5p, miRNAs associated with PDAC and chronic pancreatitis, respectively, could be used to differentiate these two diseases in combination with serum CA 19-9 [161]. The use of exosomes for PDAC diagnosis is still investigational; however, they provide a foundation for future diagnostics that can identify PDAC prior to imaging findings.

## 7. Cost-Effectiveness of Personalized Medicine

A major consideration for providing personalized approaches to cancer treatment is the cost associated with DNA sequencing to identify targetable mutations [162]. Prior to NGS, single gene testing was performed to identify known driver mutations for an individual cancer and, oftentimes, several sequential tests would be performed for multiple genes, which increased costs and time to diagnosis [163]. NGS allows for the assessment of multiple biomarkers simultaneously, thereby reducing testing costs and time to diagnosis [163]. NGS is now more readily available, and the cost has dramatically decreased in the past decade; in 2012, a test cost approximately USD 5900 (US dollars [USD]) whereas in 2021, the cost decreased to USD 454 [164]. The cost-effectiveness of NGS has been difficult to study given the lack of standardized testing guidelines across countries and institutions, variations in testing equipment and infrastructure, and heterogeneity of comparative cost studies [164,165,166]. Mirzi et al. published the most recent and comprehensive review of the available cost-effectiveness literature to date [164]. Among these studies, the authors report that cost-effectiveness was most frequently assessed by (1) direct testing costs; (2) indirect testing costs (including personnel expenses, need for rebiopsy, turnaround time, and equipment costs); and (3) comparison of long-term patient outcomes and costs associated with treatment and diagnosis. Direct testing costs were frequently reduced by using targeted panel testing of a defined gene panel ranging from 3 to 52 genes instead of sequencing the entire genome (≥30,000 genes). Analyses of both direct and indirect costs found that targeted panel testing was more cost-effective. The cost of NGS ranged from USD 250 to 7700; targeted panel testing had the lowest average cost of approximately USD 2100 per test and whole genome sequencing the most expensive, with an average cost of USD 3420. In terms of patient outcomes, most studies found that NGS testing was cost-effective at the present time, especially when a panel of genes needs to be tested, compared to testing single genes sequentially [164]. When considering both direct and indirect costs, several recent studies have demonstrated the cost-effectiveness of NGS panel testing for patients with newly diagnosed metastatic lung cancer from both a United States commercial payer system and Medicare perspective when factoring in gene panel testing, quicker time to obtaining test results, and costs associated with repeating biopsies [163,167]. From a patient outcome perspective, the cost of NGS testing was consistently found to improve quality-adjusted life-years (QALY), largely attributed to the identification of effective targeted therapies [168,169]. The major driver of medical expense was attributed to the cost of the subsequent therapy [164,169,170].

The availability of molecular testing is more difficult to quantify since it varies greatly by cancer type, clinical management guidelines, and country. While there are many commercially available molecular tests in the United States, more clinical data is needed to guide implementation of these tests in clinical practice. This data is better established in other cancer types, and with respect to PDAC is still in early stages [171].

## 8. Conclusions

Pancreatic adenocarcinoma remains an extremely aggressive disease that is difficult to diagnose at early stages where treatment is more effective. These shortcomings are due to lack of a screening test and limitations with pancreatic imaging such that there is a high false-negative rate for detection of occult metastatic disease. Ongoing efforts utilizing novel biomarkers are exciting for the early detection and prognostication of PDAC. Detection of CTCs or products of cells such as DNA or exosomes potentially allows for a more specific diagnostic tool, especially in combination with CA 19-9 levels. These modalities are limited in part by the technologic constraints of identifying scant quantities of small molecules. None of these modalities are ready for clinical use today as they are not broadly applicable; however, they pave the way for future applications. Future studies are needed to contextualize the clinical utility of these tests in treatment paradigms. With evolving molecular profiling technology, the cost of molecular testing is decreasing, and more treatment options are potentially available to patients.

## Figures and Tables

**Table 1:** Summary of biomarkers and associated clinical trials.

Type	Biomarker	Significance	Uses	Limitations	Clinical Trials
**Oncogene**	KRAS	Most common genetic alteration in pancreatic cancer (90–95%) Identified via genetic and ctDNA testing Type of KRAS mutation can guide the selection of combination therapy Targeted therapy: KRAS G12C inhibitors (sotorasib and adagrasib)	Prognostic: KRAS ctDNA is associated with lower OS and PFS and earlier disease recurrence	High innate and acquired drug resistance There is no targeted therapy for the most common type of KRAS mutation (G12D ~40%)	Phase 1–2 trial, sotorasib for advanced pancreatic cancer with KRAS G12C mutation who received prior treatment (NCT03600883) Phase 2 study, MRTX1133 for advanced solid tumors with KRAS G12D mutation (NCT05737706)
**Tumor suppressor genes**	TP53	Associated with impaired response and resistance to chemotherapy	Predictive: ~5% absolute risk of developing PDAC Prognostic: poor OS and accelerated metastasis	Targeted drug therapy is difficult due to a high diversity of mutations	None
	SMAD4	Associated with resistance to radiation therapy	Prognostic: accelerated metastasis	Difficult drug target due to variable effects of the mutation on different tumors OS data mixed	None
	CDKN2A	Targeted therapy: CDK4/6 inhibitors (palbociclib)	Predictive: >15% risk of developing PDAC	Carriers are high-risk, screening recommended No association with OS	Phase 1 trial, palbociclib with PI3K/mTOR Inhibitor (Gedatolisib) (NCT03065062) Phase 1 trial, palbociclib with ERK inhibitor (Ulixertinib) (NCT03454035)
**DNA damage repair**	BRCA 1/2	BRCA2 associated with improved OS compared to BRCA1 Platinum-based therapy OS benefit in BRCA2 mutation Targeted therapy: PARP inhibitors (Olaparib)	Predictive: ~5–10% absolute risk of developing PDAC	Carriers are high-risk; screening recommended Current screening methods are expensive and have high false positives	POLO: Phase 3 trial, maintenance therapy with Olaparib in metastatic BRCA 1 or 2 mutated pancreatic cancer (NCT02184195)
**Serum protein**	CA 19-9	Effective tool in staging; levels increase with higher stage Higher levels during laparoscopy may suggest unresectable disease Decreases correlate with response to systemic therapy Used for surveillance	Prognostic: patients who had lower levels at diagnosis and after resection had improved OS Surveillance: rising level suggests disease recurrence/progression	False normal in Lewis antigen-negative patients (10%) Poor diagnostic and screening test Levels can be elevated in benign conditions	None
**Membrane tyrosine kinase, Oncogene**	HER2	Incidence of HER2 overexpression in PDAC: ~40% Targeted therapy: anti-HER2 drug-antibody conjugate (trastuzumab deruxtecan; T-DXd)	Prognostic: HER2 overexpression suggests worse OS	T-DXd has a low treatment response in PDAC OS data mixed	DESTINY- ∘ PanTumor02: Phase 3 study, T-DXd for metastatic solid tumors expressing HER2 (NCT04482309) ∘ Lung01: Phase 2 study, T-DXd for metastatic non-small-cell lung cancer expressing HER2 (NCT03505710) ∘ CRC02: Phase 2 study, T-DXd for metastatic colorectal cancer expressing HER2 (NCT04744831)
**Transmembrane tight junction protein**	Claudin 18.2	Not expressed in normal pancreatic tissue Targeted therapy: immunoglobulin G1 monoclonal antibody (zolbetuximab)	Prognostic: Claudin 18.2 expression is associated with improved OS	Zolbetuximab is not FDA-approved for PDAC treatment OS data mixed	SPOTLIGHT: Phase 3 trial, zolbetuximab plus mFOLFOX6 for select gastric or GE junction cancer (NCT03504397) GLOW: Phase 3 trial, zolbetuximab plus CAPOX for select gastric or GE junction cancer (NCT03653507) Phase 2 trial, zolbetuximab plus nab-paclitaxel and gemcitabine as first-line treatment in metastatic pancreatic cancer (NCT03816163)
**Tumor epithelial cell**	Circulating Tumor Cell (CTC)	Identify targeted therapies using surface-antigen and genomic expression profiles Decrease in CTC levels during treatments associated with treatment response	Diagnostic: early detection possible using circulating pancreatic epithelial cell levels Prognostic: higher preoperative CTC is associated with worse OS, PFS, earlier recurrence, and suggests occult metastatic disease Surveillance: Can detect recurrence up to 2 months earlier than imaging	Rare in circulation Analysis is difficult due to difficulty isolating cells	None
**Tumor extracellular DNA**	ctDNA	Detected using liquid biopsy, less invasive than tissue biopsy Lower levels associated with early-stage or resected cancers; useful for surveillance and treatment response	Diagnostic: potential for diagnosing early-stage disease Prognostic: detection is associated with worse OS, PFS, earlier disease recurrence, higher metastatic disease burden	Very low concentration in early-stage disease makes early detection difficult	PATHFINDER: prospective, multi-center study, using cfDNA to determine the feasibility of a multicancer early detection (MCED) blood test (NCT04241796) PATHFINDER 2: prospective, multi-center study, determining the safety and performance of the MCED blood test from PATHFINDER (NCT05155605)

## Abbreviations

The following abbreviations are used in this manuscript:

PDAC	Pancreatic ductal adenocarcinoma
CTCs	Circulating tumor cells
RNA	Ribonucleic acid
DNA	Deoxyribonucleic acid
OS	Overall survival
HR	Hazard ratio
MMR	Mismatch repair
MSI	Microsatellite instability
CA 19-9	Carbohydrate antigen 19-9
MCNs	Mucinous cystic neoplasms
IPMNs	Intraductal papillary mucinous neoplasms
PPV	Positive predictive value
NPV	Negative predictive value
AUROC	Area under receiver operating characteristic curve
IQR	Interquartile range
PCR	Polymerase chain reaction
NGS	Next generation sequencing
USD	US dollars
QALY	Quality-adjusted life-years
